# Remarkable Case of Various Unsuspected Lesions, Ending in Atrophy

**Published:** 1829-07-01

**Authors:** 


### Case of Hypertrophy of the Left Ventricle—Diminution of Capacity in the Right Ventricle—Extensive Induration of the Arterial System—Softening of the Brain—Old and Extensive Ulcers in the Ileum—Death from extreme Atrophy. By James Johnson, M.D.

The following highly interesting and melancholy case is calculated to teach humility—to inculcate charity—and to moderate pride, as far as the medical art and its professors are concerned.

The subject of the case was a young officer, about 25 years of age. He had been serving with his regiment, in a part of continental Europe by no means unhealthy, in the year 1827, and was then apparently in good health. It is probable, however, that he may have lived rather freely about that period, and for some time previously.

In the Autumn of the above year, and without any apparent cause, or deviation from usual health and habits, he was suddenly affected, after some riding exercise, with paralysis of the right arm and leg, without any loss of consciousness, or any intellectual disturbance. He was lying on a sofa when the surgeon arrived, who asked him what was the matter, as he appeared in good health? The answer was, "don't you see that I am paralytic." The paralysis, however, went off in about six hours from the commencement, without bleeding, but only with some aperient medicine. The next day he was bled, in consequence of some threatenings of a return. He had no more paralytic affection during the remainder of his life—upwards of 18 months—and he did military duty on his voyage home, in the year 1828. As far as could be collected from the patient and from the medical attendants, there had been derangement of the digestive organs, both before and ever after the above attack. Be this as it may, gradual and regularly progressive emaciation of the body went on from this time till the day of his death, (24th of May, 1829) with the exception of a few weeks, which will be presently noticed.

After his return to England, he was under the care of a surgeon in London for a local complaint—and afterwards he was attended occasionally in Yorkshire by another medical gentleman. What were the opinions of these practitioners cannot now be exactly ascertained. Some of them, at least, viewed the complaint as that of disordered function of the stomach, liver, and bowels. The patient was able to take some considerable degree of exercise, amounting even to hunting and shooting, in the country, during the Autumn and Winter of 1828. According to his own account, the progress of emaciation was, for some time, suspended in the country.

In the month of February, 1829, the gentleman presented himself to me, at my own house, and the degree of emaciation which then obtained instantly raised in my mind the idea of mesenteric disease. After giving a very slight history of his complaint, he observed that the essential and distressing symptoms were, "a constant fermentation in his stomach, with acid eructations, flatulence, sense of distention in his bowels, progressive emaciation and debility." He was stripped, and examined in my own library. The emaciation was, indeed, alarming. On laying him on a sofa, the abdomen was so extenuated, that I could feel the aorta from the cœliac artery to its bifurcation. I thought I felt enlarged mesenteric glands, and my first impression was, that mesenteric disease obtained. The chest sounded well in all parts, and the respiration was loud and distinct throughout the whole thorax. The heart beat strongly, but not over a larger space than usual—the pulse was 80 and weak. There was no disturbance of the intellectual functions, nor of any of the senses—the appetite was rather craving than otherwise—and the bowels were reported to be irregular, sometimes relaxed, sometimes confined. The tongue was moist, and slightly furred at the root. The patient had been losing weight, at the rate of from three to five pounds weekly, for a considerable time previously, and the cravings of nature were such, that he took more nutriment, in the aggregate, than a man in health.

As I said before, the impression on my mind was, that the patient laboured under mesenteric disease. A mild aperient was prescribed, and again he presented himself. On examination, no mesenteric glands could be felt, and he informed me that he had passed some lumpy stools. I prescribed mild aperients, and a bitter infusion, with a very small proportion of the sulphate of quinine. I advised him to moderate the quantity of his food; but, on this point, he seemed determined to be the guide of his own conduct. During the first week of this plan the progress of emaciation stopped, and he lost but a quarter of a pound. During the preceding two or three weeks he had lost from four to five pounds weekly. In a week or two more, the "fermentation in his stomach," which was the great source of his grievances, almost entirely ceased;—but, upon the whole, the emaciation advanced. As the patient now made known his residence, I had an opportunity of examining the state of the evacuations from the bowels. They were exceedingly variable—sometimes light or clay-coloured—sometimes dark and lumpy—sometimes slimy—sometimes entirely healthy and formed—but, generally speaking, fetid in smell, and deviating from a state of health. The pulse hardly ever varied from 80 in number, being weak, small, but regular. I candidly confess—and I confessed to the relatives of the patient at the time, that I was at a loss to form any accurate knowledge of the nature of the disease. I did the best that my judgment prompted for checking its progress, but I was unsuccessful. My avocations prevented me from keeping notes of the case; but I think it was about the beginning of April that another physician was called into consultation. He was equally at a loss to form any thing like a determined diagnosis of the disease. We both suspected that some mischief had been going on in the brain from the period of the paralytic seizure in Portugal; but it appeared to us quite evident, that the immediate cause of the progressive emaciation and atrophy was in the digestive apparatus.* Dr. —— recommended an increase of the quinine (to the amount of four or five grains in the day) with alterative aperients. On this plan, to the surprise of the patient, as well as of ourselves, he gained, in 14 days, 7¼ lbs. in weight—and began to drive out daily in his cabriolet. In the succeeding week, he lost 5½ pounds! He thus went on, sometimes stationary, but generally losing flesh and strength, his appetite continuing tolerably good, and the evacuations from the bowels frequently healthy, but more frequently otherwise.

One evening, in the latter end of April, he sat at the window of a drawing-room while the easterly wind prevailed, and got a chill. He next day complained of pain, or at least uneasiness, in the back part of his head, and there was an acceleration of pulse, with some febrile symptoms. The bitter tonics were discontinued, and some saline febrifuges prescribed. As the head-symptoms rather increased, we had the patient cupped on the back of the neck, and kept in bed. About this time, my colleague, on examining the abdomen, thought he felt something like a tumour pulsating near the origin of the renal arteries, and mentioned this suspicion to the parent of the patient. A surgeon was added to the consultation; but nothing could be detected in the abdomen. As the pulse was quick and hard, and the patient complained of some uncomfortable sensations about the head, we agreed that six ounces of blood should be drawn from the arm. It was much buffed and cupped. In the course of the same night, *the region of the left parotid gland became swelled and inflamed*. Next day he complained of an indescribable sensation of distress whenever he attempted to turn himself from

* The pulse, when Dr. —— was called in, and during the whole of the Doctor's attendance, till the time of the patient's taking cold at a window, and till the commencement of cynanche parotidea, was weak. The expression which Dr. — used was—" this is a shabby pulse."

one side to another in bed. While the three medical attendants were at the bed-side, and while he attempted to turn himself, he went off into a most tremendous convulsion, in which he nearly expired. During the fit, we drew off about 16 ounces of blood, (which presented scarcely any appearance of inflammation,) the pulse being hard and 150 in the minute. After being, for some time, on the verge of death, he suddenly opened his eyes, and, in a few minutes, was in the same condition as before the fit. The left side of the face now became much swelled, the parotid gland was apparently inflamed, and fears were entertained that this would be a most formidable complaint. Rigid abstinence and the antiphlogistic regimen were enjoined—the inflammation proceeded to suppuration—the part discharged healthy pus—healed—and was found not to invo.ve the parotid gland, but only the neighbouring cellular structure. The pulse, after the suppuration, fell back to nearly its original state, excepting that it was quicker, being from 90 to 100. Mean time, the unfortunate patient emaciated with increased rapidity; and for eight or ten days before his death, stimulants—quinine in larger quantities than he had ever before taken—and every kind of wine and nutriment that he fancied, were allowed. He died, or rather ceased to exist, on the 24th of May, preserving every sense and every intellectual faculty to the last moment. Such a picture of atrophy was perhaps never before witnessed. Yet the cravings of Nature were urgent, and he ardently begged for nutriment during the time he was on the rigid plan of abstinence for (supposed) inflammatory action in the brain. During all this time, there was not a single symptom that could have fairly authorised a suspicion of ulceration of the intestines. Neither was there any unequivocal symptom of cerebral affection. The history of the case—the primary paralytic seizure—the convulsion fit—and some tingling sensations in the right arm, were the only data on which to ground the supposition of cerebral disease. Till within three days of the patient's death, every nerve of sense was perfect in its function—and till within an hour of his death, the intellectual faculties were in a state of integrity. For the two last days, sight was very imperfect, and the left eye was turned upwards and outwards.

Sectio Cadaveris, 25th May, 1829.

The body was examined, 24 hours after death, in the presence of several medical gentlemen. The body was emaciated to the utmost possible degree.

*Cranium.* The calvarium was attached pretty firmly to the dura mater.

On removing it the latter membrane presented no marks of inflammation or increased turgescence; on the contrary, it was, if any thing, paler than natural. Between the tunica arachnoides and pia mater some serous fluid was effused, which escaped, in great measure, on wounding those membranes. The tunica arachnoides was not itself opaque, nor were the vessels of the pia mater loaded, but rather the reverse.

The hemispheres were sliced down to the level of the corpus callosum, without our observing any thing unusual. The arteries of the latter part, however, presented a morbid alteration of their coats, opaque patches of gritty feel and hard consistence. No red points were observed on the sections of the brain, which was very pale.

The lateral ventricles contained about an ounce of clear limpid serum. The plexus choroides on each side was blanched and pale; and, entangled, as it were, in the substance of either, was a mass of curdy, atheromatous-looking matter, oblong in shape, and broader than a pea in diameter.

The brain was now gradually sliced down to its basis. It was generally softened, or in the state of "ramollissement." Its arteries were extensively affected with the same morbid condition as those of the corpus callosum, which were first noticed.

The part of the anterior lobe of the left hemisphere, resting in the left orbital fossa, appeared to be softer than the rest. The same thing was thought to be the case with the pons varolii. The cerebellum was also softened like the cerebrum.

The pulpy part of the olfactory nerves, resting on the cribriform lamella of the æthmoid bone was very soft. The arteries at the basis cranii, viz. the basilar, vertebral, &c. partook of the same state as their ramifications had done in the brain.

*Thorax.* The pleural cavities and the lungs were perfectly free from disease. The pericardium was unaltered, and no fluid was contained within its cavity.

The heart was not so healthy; its coronary arteries had hard cartilaginous patches in their coats. The heart, on the whole, was a little enlarged in its calibre. The auricles shewed nothing particular, but not so the ventricles. The right was not attenuated in its parietes, but its cavity was surprisingly small. The walls of the left ventricle, on the contrary, were an inch, and in some parts upwards, in thickness, and the cavity was proportionally much greater than that of the right. The mitral valve presented osseo-cartilaginous depositions, near its attached border, and the same existed in the semilunar valves, at their origin from the sides of the aorta.

The arch of the aorta and its thoracic portion appeared to be sound.

*Abdomen.* The small intestine had externally a curiously black and mottled appearance. There was no extravasation into the cavity of the peritonæum, but about the centre of the intestinum ileum, was noticed externally a kind of circular brown stain, in the centre of which, was a perforation of all the coats, somewhat larger than a pin's head. The gut was laid open, and a circular ulcer of the mucous and muscular tunics was discovered at this spot, as large in circumference as a shilling. The peritoneal coat alone remained, and even this had in one spot given way, forming the circular aperture described.

Ulcerations of this kind, mostly circular, some affecting only the mucous coat, others the mucous and muscular, and denuding the peritoneal, formed a kind of chain, extending for 12 or 15 inches along the interior of the gut. In other places the mucous membrane presented patches of acute inflammation, unaccompanied by ulceration. As we approached the ileo-cæcal valve, the intestine resumed its natural appearance, and the latter part with the ascending arch of the colon were healthy.

The liver was sound; so was the spleen; and the stomach also. The kidneys were diminutive, and not exactly healthy in their structure. The renal capsules, especially the left, were remarkably large, and their coat, which was of a yellowish buff colour, was full a line in thickness, and corrugated on its surface.

The abdominal aorta disclosed, when cut into, some faint streaks of atheromatous deposition, but the mesenteric vessels and the renal shewed them more distinctly. The iliac vessels were perfectly sound.

The mesenteric glands were slightly enlarged.

The bladder was full of urine.

(Signed) HENRY J. JOHNSON.

On the next day I wrote a note to Dr. Elliotson, (who had witnessed the examination) inclosing the minutes of the dissection, and requesting to be favoured with any remark he had to make on the statement. The following was his reply.

"My dear Sir,

"The account appears very correct. I did not handle the brain; but though I hardly think it was generally soft, it was undoubtedly so at the septum lucidum, fornix, and over the left orbitar plate of the frontal bone. *The softening was clearly not the result of inflammation, (for I never saw so pale a brain and membranes,) but either of deficient distribution of blood, from the diseased state of the arteries, or of the cachectic disposition which gave rise to this diseased state, and to the depositions in the plexus choroides.*" J. ELLIOTSON.

Grafton Street, Thursday, 28th May, 1829.

I have now to entreat the reader's attention to the highly interesting phenomena presented on dissection. A careful review of the post-mortem appearances lead me to class them in the following order of precedency. I think it will be admitted by those who are conversant with pathological investigations, that the consolidation and thickening of the left ventricular parietes, and the induration of the arterial coats, were first in order of occurrence. That these changes had *commenced* long before the attack of temporary paralysis, no rational doubt can be entertained. The consolidation of the heart, as compared with its size, was greater than I had ever before seen. It felt like a lump of lead when poised on the hand. Have we any marks of inflammation here? The pericardium was beautifully pellucid—no effusion—no vestige of coagulable lymph. This hypertrophy of the left ventricle, with encroachment on the right—this condensation and increase of structure are produced by causes of which we are ignorant. The same may be said of the atheromatous deposition between the tunics of the arteries. Is this too an inflammation, and to be cured by copious blood-letting?

We next come to the softening of the brain. Without entering into the litigated dispute respecting the causes of "ramollissement," it would be absurd to ascribe it, in this case, to inflammation. *There was not a single trace or evidence of inflammation in the brain.*

> "De non apparentibus et non existentibus, eadem est ratio."

If, indeed, we had found a portion or portions of brain softened down, with a surrounding injection of vessels and the other well-known products of inflammation, we might fairly perhaps conclude that a process of inflammation had been going on. But in the case before us, there was not one iota indicative of inflammation—but quite the reverse. The brain was in as complete a state of atrophy (in its way) or *innutrition*, as the body of the unfortunate deceased. On this point every unbiassed pathologist must coincide with Dr. Elliotson.

At whatever period, then, the softening of the brain commenced, we have no evidence that there ever was inflammation there. The temporary paralysis disappeared without blood-letting—and the emaciation went on from that time. There is not therefore the slightest reason to think that copious depletion, at the time of the first attack, would have had any influence over the progress of the disease. It would not have stopped the hypertrophy of the heart—nor the ossification of the arteries—nor the softening of the brain, had such a process then commenced. The surgeon who attended the patient therefore, in the first instance, is completely absolved from censure.

The state of the heart deserves particular attention. Here we have the parietes of the left ventricle immensely thickened, and prodigiously consolidated, at the expense of the right ventricle, whose capacity was diminished to one half its natural size. I heard it remarked that, in this case, "the ossification of the arteries was the *consequence* of the hypertrophy of the heart—Nature having taken this method of guarding against the great impulsion from the central organ of the circulation." This I conceive to be most erroneous pathology. Nature could not take a worse mean of strengthening arteries than by depositing calcareous or cartilaginous, or atheromatous matters between their coats. It is just in such places, that the arteries give way. A sounder pathology teaches us to view the induration of arteries as the cause rather than the consequence of hypertrophy of the heart. Indurated arteries lose, with their elasticity, the power of assisting the heart in the circulation of the blood:—the heart is therefore called on for greater exertion. It is probable, however, that these two states of the heart and arteries, have no necessary connexion with, or dependence on each other, since we so often see them separate. But be this as it may, the dependence of arterial ossification on cardiac hypertrophy, is clearly out of the question.

It was said, in this case, that the left ventricle must have possessed great muscular force. I very much doubt this inference. Hypertrophy is itself a disease, and I doubt whether a thickened and diseased ventricle has more power to circulate the blood than a healthy one. True, an enlarged and consolidated heart will beat with more force against the ribs; but we very often find, under these circumstances, that the pulse is feeble. Such was the case during the whole of my attendance and that of the other physician, on the present patient, up to the time when cold was caught at a window, and the local inflammation of cynanche parotidea set up. After the suppuration was over, the pulse fell back to nearly its original state. I would account for this feebleness of pulse with a hypertrophied ventricle in the following manner. In the case just recorded, it was allowed by all present that the *right* ventricle could not contain more than half the usual quantity of blood, from the great encroachment on it of the *left* ventricular parietes. Now as the two ventricles act at the same time, and as the blood which feeds the left heart is supplied by the right, through the channel of the pulmonary vessels, so the quantity ejected from each ventricle during a single pulse, or during any given time, must be precisely the same. In other words, the left ventricle can only propel into the aortic system, at each contraction, the same quantity which the right ventricle propels into the pulmonary system. It is physically impossible that it can be otherwise. In the case before us, then, the left ventricle, however thickened in parietes and enlarged in capacity, could only eject, at each contraction, about half the usual quantity of blood—for this plain reason, that there was only that proportion furnished by its diminished coadjutor on the right side. And this view is confirmed by the state of the pulse in the present case. I grant that in hypertrophy of the left ventricle, and diminution of the right, there may be a greater *impulse* given to the aortic jet of blood—but the quantity thrown out, must always be in accordance with the diminution of capacity in the *right* chambers of the organ. In the present case, the brain, so far from being threatened with an overflow of blood, could only have received about half the usual quantity at each stroke of the heart.

May not this view of the case account for those intermissions of the pulse which we often find in hypertrophy of the left ventricle, and diminution of the right, where there is no valvular disease. The imperfect supply of blood from the right side, may cause imperfect ventricular contractions in the left. In the case under consideration, have we not some clue to the atrophy both of brain and body, from the diminished quantum of blood propelled from the heart at each ventricular contraction? The whole mass of blood could not have gone the round of the circulation in less than double the usual time, unless the number of pulsations in the minute should be increased in proportion to the diminished quantum moved forward at each impulse. Thus sanguification of the chyle, and arterialisation of the blood, went on at a prodigiously decreased pace in the lungs, in consequence of the diminished capacity of the right ventricle.

But there was certainly in this case another cause of atrophy which, like the former, entirely eluded the scrutiny of all the medical attendants—the ulcerations in the ileum. At what period these ulcerations commenced can only be matter of conjecture. There can be little doubt, however, of their having existed for many months—and consequently long before the patient came under the care of those who attended him to the end. There certainly was much irregularity of the bowels—much variety in the appearance of the motions—a frequent desire in the patient to take opening medicine, from a feeling of great discomfort whenever there was any thing approaching to an accumulation—and lastly, the alvine evacuations had almost always a fetid smell. But these symptoms are often present where there can be no suspicion of intestinal ulceration. The locality of these ulcerations, in the middle of the ileum, the freedom from disease in the colon, or caput coli, may have tended much to obscure the malady, and to prevent diarrhœa, the most constant of all symptoms in ulceration of the bowels. The constant discomfort of stomach was clearly referrible to the disease in the ileum.

That the general excitement, after exposure to cold, the strange sensations about the head at that time, the buff on the first six ounces of blood, were all to be attributed to the rising cynanche parotidea, no unbiassed mind can doubt. That any inflammatory affection of the brain existed at that period, is negatived to a demonstration by the dissection.*

To what are we to attribute the violent attack of convulsion after the first bleeding?—To any thing, I think, but fulness of blood! The old notions of a "rush of blood to the brain," cannot now be listened to. *Irritation* is a more feasible solution of convulsion, than inflammation or congestion—and, in the present case, the latter states are out of the question. Great losses of blood will cause convulsions in strong people—and small losses may do the same, under opposite circumstances. The whole case which I have narrated, is one of extreme interest—and for all failures of diagnosis, I make myself alone responsible, by omitting the names of my colleagues.† One thing is certain, that the primary and secondary diseases were beyond the power of art, and that the ulceration of the intestines, could it have been detected, would, in all probability have been indomitable, independently of the other lesions. The rally which the constitution made in one fortnight, during which the patient gained seven and a quarter pounds in weight, under the administration of quinine, is not a little remarkable. This rally could only have been through the medium of the digestive apparatus, improved in function by the tonic. Under more auspicious circumstances—with less disease of the vascular system, and a more energetic sensorial influence, the intestinal ulcerations might have healed. Confidence of recovery never forsook the gallant patient, or ceased to inspire hopes among his afflicted relatives, till the last. The day before his death, he was busy in planning a journey to Brighton, and that without the slightest approach to delirium!

O blindness to the future wisely given!

I have laid the facts of this melancholy case before my professional brethren, with scrupulous fidelity—and for the inferences I am solely responsible. There are some circumstances connected with it which I may touch upon at a future period.—J. J.

* Whoever has felt the confusion of thought and distressing sensations about the head in a common feverish cold with sore throat, will readily allow that all the symptoms in the present case about the head, were explicable by the cynanche parotidea, and its symptomatic fever. J. J.

† I take shame to myself for not having paid more attention to the heart. I did notice the strong impulsion of this organ, as compared with the feeble pulse; but the rapid emaciation, and the evident disorder of the stomach and digestive apparatus, drew off my attention from the central organ of the circulation. Could the actual condition of this organ have been ascertained, we had no power over the changes that had taken place. This, however, is no excuse for not having made myself acquainted, if possible, with every lesion that was capable of detection.

